# Selective detection of N6-methyladenine in DNA *via* metal ion-mediated replication and rolling circle amplification†
†Electronic supplementary information (ESI) available. See DOI: 10.1039/c6sc02271e



**DOI:** 10.1039/c6sc02271e

**Published:** 2016-08-10

**Authors:** Tingting Hong, Yushu Yuan, Tianlu Wang, Jingwei Ma, Qian Yao, Xiaoluan Hua, Yu Xia, Xiang Zhou

**Affiliations:** a College of Chemistry and Molecular Sciences , Key Laboratory of Biomedical Polymers of Ministry of Education , The Institute for Advanced Studies , Wuhan University , Wuhan , Hubei 430072 , P. R. China . Email: xzhou@whu.edu.cn ; Fax: +86-27-68756663 ; Tel: +86-27-68756663

## Abstract

N6-methyladenine (6mA) is reported as a potential epigenetic marker in eukaryotic genomes. However, accurate identification of the location of 6mA in DNA remains a challenging task. Here, we show that Ag^+^ can selectively stabilize the A–C mismatch and efficiently promote primer extension. In contrast, the complex of 6mA–Ag^+^–C is instable and therefore cannot be recognized by DNA polymerases, resulting in the termination of primer extension. Based on this finding, we successfully identified and quantified 6mA at the single-base level through the analysis of gel bands of extended primers and fluorescence measurements combined with rolling circle amplification. The high selectivity and sensitivity of this strategy may provide a new platform for the efficient analysis of 6mA in DNA in the future.

## Abstract 2

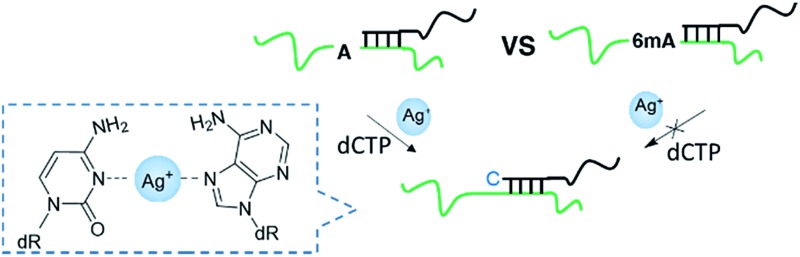
6mA can be discriminated from A in DNA due to the remarkable difference in stability between A–Ag^+^–C and 6mA–Ag^+^–C.

## Introduction

Beyond the four canonical nucleobases, 5-methylcytosine (5mC) and N6-methyladenine (6mA) have been revealed as heritable epigenetic modifications in genomic DNA.^1^ These modifications are associated with various biological functions that make them dominant epigenetic markers in diverse species. Unlike 5mC, which is abundant in eukaryotes, particularly in mammals,^2^ the genomic distribution of 6mA is initially reported to be limited to bacteria and some lower eukaryotes, and the base is found to be involved in the regulation of DNA mismatch repair, chromosome replication, cell-cycle regulation, transcription and virulence.^3^ However, recent discoveries have revealed potential roles of 6mA in regulating gene expression in several higher eukaryotes^4^ or even in vertebrates^5^ and mammalian embryonic stem cells,^6^ making it a potential epigenetic marker in eukaryotic genomes.

The previous failure to detect 6mA in higher eukaryotes is mainly due to the lack of available methods. Epigenetic DNA modifications do not alter the complementary base pairing, and to date, 6mA has never been reported to be susceptible to chemical modifications, whereas 5mC can be detected *via* bisulfite treatment, which specifically converts cytosine into uracil and leaves 5mC intact, transforming the epigenetic information into sequence differences. All of these factors contribute to the failure of standard hybridization-based methods for the detection of 6mA in DNA. To surmount this challenge, several methods based on selective polymerase,^7^ sensitive restriction enzymes^8^ and the distinct stabilities between A/6mA–G base pairs^9^ have been developed for the detection of 6mA in DNA. However, both the selectivity and sensitivity of some methods require further improvement.

Some previous studies have successfully demonstrated the application of mismatched base pairs to the direct detection of epigenetic modifications.^10,11^ In addition to the mismatches caused by non-Watson–Crick hydrogen bonding,^12^ metal-mediated base pairs stabilized through the formation of T–Hg^II^–T, C–Ag^I^–C, A–Ag^I^–C, C–Ag^I^–T and 7-DeazaA–Ag^I^–T base pairs are also reported,^13^ in which metal ions are coordinated to the nitrogen donors on the rings of these nucleosides. As an alternative type of artificial base pairs, these metal ion-stabilized base pairs could also be further recognized by DNA polymerases, resulting in the incorporation of a mismatched deoxynucleotide into DNA primers through replication. However, the interactions between metal ions and epigenetically modified nucleoside-containing base pairs^14^ are not well studied, especially for N6-methyladenine.

**Fig. 1:**
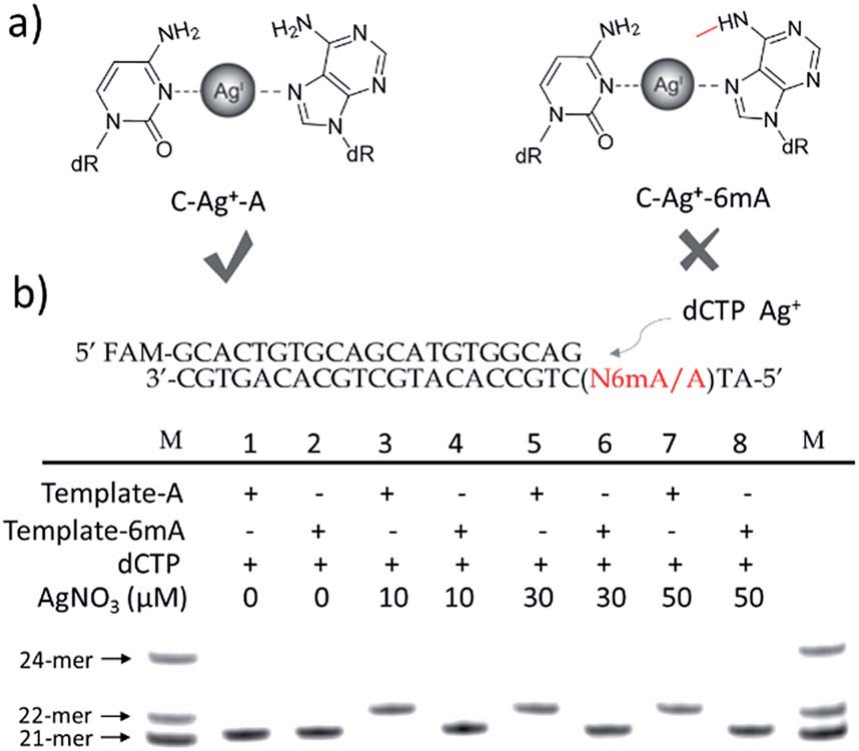
Discrimination of 6mA from A in DNA templates through Ag^+^ mediated replication by Klenow fragment (KF exo-) DNA polymerase (a) structure difference of Ag^+^-mediated base pairs between A/6mA–Ag^+^–C. (b) Sequences of A/6mA-containing templates and FAM-labeled primer, and the effect of Ag^+^ on dCMP incorporation efficiency for A and 6mA-containing templates through single nucleotide incorporation reactions. M indicates the marker for the 21-*mer*, 22-*mer* and 24-*mer* primers.

We supposed that the introduction of 6mA might interfere with the formation of a metal ion-mediated mismatch because of the steric hindrance caused by the methyl group at the N6 position of adenine (Fig. 1a), which may further disturb recognition by DNA polymerases. Inspired by this potential difference between 6mA and A in metal ion-triggered replication, we here developed a new method for the site-specific detection of adenine methylation in both ssDNA and dsDNA using Ag^+^.

## Results and discussion

### Termination of primer extension caused by 6mA in DNA

We chose two 24-*mer* templates containing A or 6mA as DNA targets, with identical sequences except for one nucleotide difference (A/6mA). To investigate the efficiencies of dCMP incorporation triggered by Ag^+^ for A/6mA-containing templates, we first performed single nucleotide incorporation reactions using Klenow fragment exo- (KF exo-) DNA polymerase.

In the absence of Ag^+^ and dTTP, no incorporation of dCMP into primers opposite either A or 6mA in DNA templates was observed (Fig. 1b Lane 1–2). With the addition of Ag^+^, however, the 21-*mer* primer was gradually extended to a 22-*mer* due to the formation of A–Ag^+^–C with increasing elongation time (Fig. S2†). To our delight, our data demonstrated a strikingly different result for the 6mA-containing template compared to the corresponding template containing A. The elongation reaction was completely terminated at the site opposite 6mA in the template, and few extended primers were detected, even with increased reaction time. These results indicated that the 6mA–C mismatch may not be stabilized by Ag^+^ and further recognized by KF exo- DNA polymerase.

Next, we performed primer extensions for the A- and 6mA-containing templates at various concentrations of Ag^+^ (from 0.5 μM to 10 μM, Fig. S3,† from 10 μM to 50 μM, Fig. 1b), DNA Klenow fragment exo- (from 0.1 unit to 1 unit, Fig. S4†) and dCTP (from 10 μM to 100 μM, Fig. S5†) to explore the potential recognition of 6mA–Ag^+^–C by DNA polymerase. Here, the same pausing effect on elongation reaction was observed for 6mA-containing templates under all conditions tested, which showed the high selectivity of dCMP incorporation for the A-containing template in this Ag^+^-mediated replication. Thus, this significant pausing effect of 6mA may provide a new platform for the site-specific detection of adenine methylation in DNA.

### Steric hindrance caused the instability of the 6mA–Ag^+^–C mismatch

**Fig. 2:**
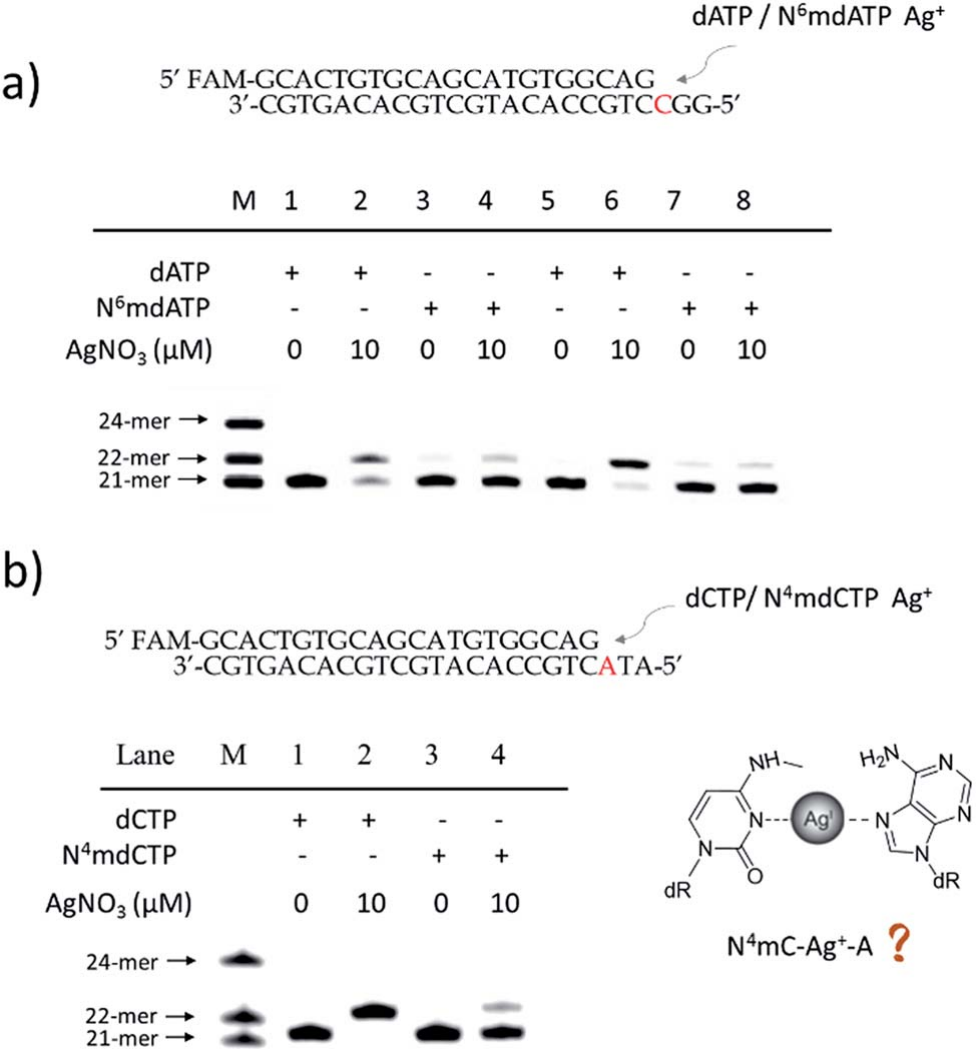
(a) Incorporation efficiencies of dAMP and N6mdAMP opposite C residues in DNA templates for different incubation times in the absence or presence of Ag^+^. The incubation time for Lane 1–4 and Lane 5–8 is 5 min and 10 min, respectively. The 21-*mer* primers were completely extended into 22-*mer* with the addition of dATP and Ag^+^ after a 10 min incubation at 37 °C. Compared with the high incorporation efficiency of dAMP, much fewer extended primers were detected with the addition of N6mdATP. (b) Incorporation efficiencies of dCMP and N4mdCMP opposite A residue in DNA templates in the absence or presence of Ag^+^. The reactions were conducted under the same conditions described above for 30 min. Compared with the incorporation of dCMP, a much lower efficiency of N4mdCMP incorporation was measured. M indicates the marker for the 21-*mer*, 22-*mer* and 24-*mer* primers.

To further verify the difference between A–Ag^+^–C and 6mA–Ag^+^–C mismatches in this metal ion-mediated replication, we firstly investigated the incorporation of dAMP and N6mdAMP triggered by Ag^+^ opposite C residues in DNA templates (Fig. 2a). Similarly, dAMP was successfully incorporated into the primers with the aid of Ag^+^. As expected, considerably much fewer extended primers were detected with the addition of N6mdATP, indicating that the 6mA–C mismatch may not be stabilized by Ag^+^ regardless of whether 6mA served as the incoming deoxynucleotide or residue in the DNA templates. The higher incorporation efficiencies of dAMP and N6mdAMP compared with dCMP were caused by the lower energy required for the *anti* to *syn* rotation of the incoming dATP and N6mdATP.^13*c*^

We supposed that the main reason for the instability of the 6mA–Ag^+^–C mismatch was the steric hindrance caused by the methyl group on the N6 position of adenine, which further resulted in the failure of recognition by DNA polymerase. To verify this potential steric effect, we removed the methyl group from 6mA and added it to the N4 position of cytosine to see if this methyl group can still reduce the stability of the 4mC–Ag^+^–A mismatch and cause the same pausing effect in this Ag^+^-mediated replication. As expected, much lower incorporation efficiency was detected for N4mdCMP compared to dCMP (Fig. 2b). We concluded that the introduction of the methyl group at the N4 position of cytosine and the N6 position of adenine might block the coordination between Ag^+^ and nitrogen donors on the rings of these nucleosides, resulting in the instabilities of 6mA–Ag^+^–C and 4mC–Ag^+^–A. Thus, not only N6-methyladenine (6mA) but also N4-methylcytosine (4mC), another important epigenetic modification in the genomic DNA of bacteria,^15,16^ can be identified through this metal ion-mediated replication.

By contrast, the introduction of a methyl, hydromethyl, formyl or carboxyl group at cytosine (5mC, 5hmC, 5fC and 5caC) did not interfere with the formation of Ag^+^ stabilized base pairs (Fig. S6†) because these modifications at the C5 position of cytosine cannot cause a similar steric-hindrance effect as 6mA and 4mC do. We also tried to use Ag^+^ to stabilize N1mA–C but failed to achieve the extended primer although the methyl group at the N1 position did not have a disturbing effect on the coordination between Ag^+^ and nitrogen donors (Fig. S7a†). We speculated that this termination may be caused by the electrostatic repulsion between Ag^+^ and the positively charged N1mA. Nevertheless, N1mA could still be distinguished from 6mA and A owing to the position of its methyl group at the Watson–Crick interface, which can stall the incorporation of dTMP^17^ (Fig. S7b†). Thus, besides the steric-hindrance effect, electrostatic interaction should also be taken into consideration for the Ag^+^ stabilized base pairs.

### Quantitative evaluation of the degree of adenine methylation

**Fig. 3:**
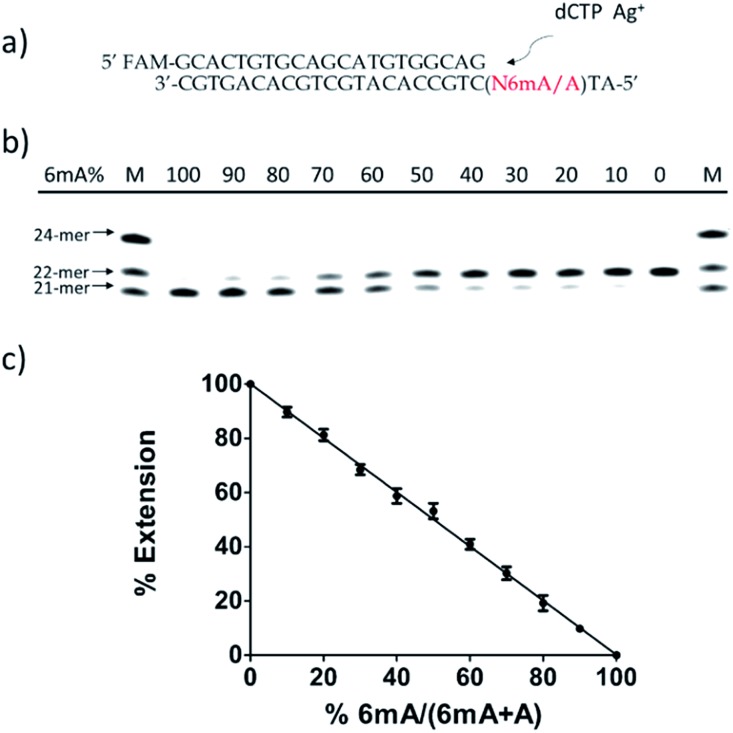
Quantitative evaluation of the degree of adenine methylation in DNA through dCMP incorporation at specific sites. (a) Sequences of A/6mA-containing templates and FAM labeled primer. (b) 20% denaturing PAGE analysis of the primer extensions for the templates with various ratios of 6mA. (c) Linear relationship between primer extension percentages and methylation levels. Error bar, mean ± SEM, *n* = 3.

Then, we tested whether this disparate incorporation efficiency of dCMP for A and 6mA templates could be employed for the quantitative evaluation of the degree of adenine methylation. First, various ratios of 6mA-containing templates were mixed with their A-containing counterparts to mimic samples with diverse methylation levels. We measured the yields of extended primers under the optimized conditions described above (Fig. 3b). The extension percentage showed a linear relationship with the methylation level (Fig. 3c). This quantitative result demonstrated the successful application of this metal ion-mediated replication to the quantitative evaluations of the adenine methylation level at specific sites.

### Metal-ion mediated replication using other series of templates, DNA polymerase and metal ions

**Fig. 4:**
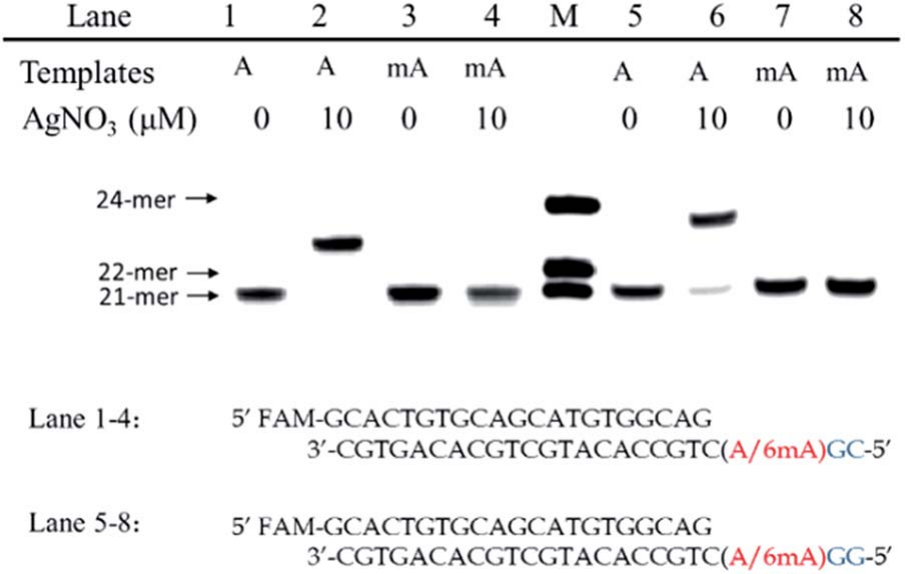
The discrimination of A and 6mA containing templates (A/6mA–GC and A/6mA–GG) through Ag^+^-mediated full-length elongation.

Furthermore, we also tested two other series of A/6mA templates, in which the last two bases were changed to GG/GC (Fig. 4), to determine whether this metal ion-mediated replication would discriminate 6mA from A in a similar way. With the addition of Ag^+^ and dCTP, the primer was extended into a 23-*mer* for the A–GC template and was fully extended into a 24-*mer* for the A–GG template with high efficiency, indicating that the incorporation of dCMP opposite A in templates did not affect the full-length elongation. However, the KF exo-polymerase still failed to catalyze the primer extensions for the 6mA containing templates.

In addition, Taq DNA polymerase was used to investigate whether this extension difference between A and 6mA is specific to KF exo- polymerase in this metal ion-mediated replication. Fig. S8 and S9 illustrated a similar efficient extension and inhibition for the A and 6mA templates, respectively, indicating the highly disparate stabilities of A–Ag^+^–C and 6mA–Ag^+^–C complexes in primer extensions.†

To investigate the effect of other metal ions on 6mA discrimination using this metal ion-mediated assay, the extension efficiencies of single-nucleotide incorporation and full-length extension using A/6mA–TA and A/6mA–GG templates, respectively, were both conducted in the presence of Ag^+^, Li^+^, Cu^+^, Cu^2+^, Zn^2+^, Pb^2+^, Ni^2+^, Mn^2+^, Co^2+^, Fe^2+^, Fe^3+^ or Al^3+^ (Fig. S10 and S11†). The results demonstrated that some other metal ions can catalyze primer extensions for A template to a certain degree, while the greatest extension difference between the A and 6mA templates was observed for Ag^+^.

### Discrimination of 6mA from A in dsDNA templates

**Fig. 5:**
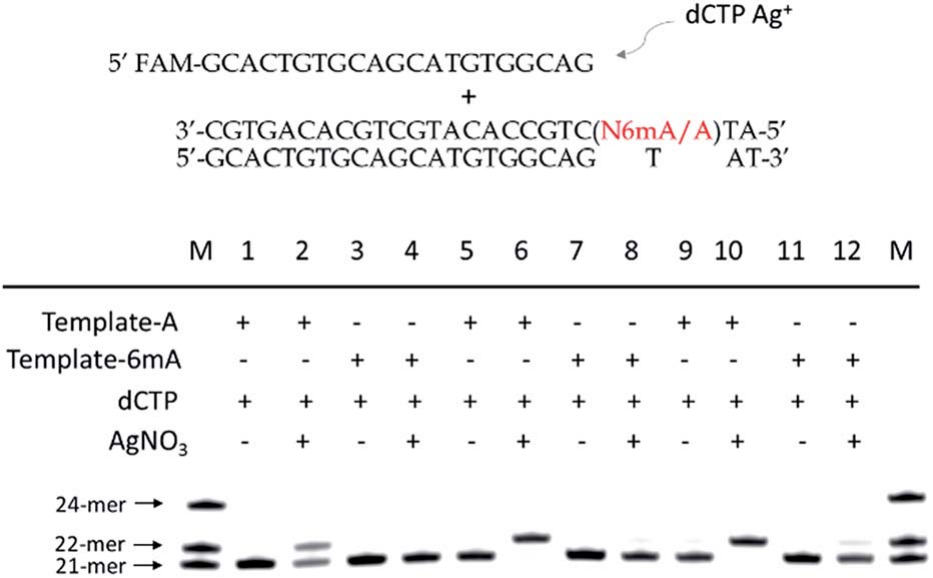
Discrimination of 6mA from A in double-stranded DNA with the aid of Ag^+^ using Taq DNA polymerase. With increased cycles (denaturation at 95 °C for 15 s, annealing and replication at 68 °C for 10 min), tenfold primers were completely extended into 22-*mer* for A-containing dsDNA templates in the presence of Ag^+^, while nearly no extensions were observed for 6mA containing dsDNA (10 cycles for Lane 1–4, 15 cycles for Lane 5–8 and 20 cycles for Lane 9–12).

As 6mA naturally exists in double-stranded DNA, we further tested whether our metal-ion mediated replication could also be applied for dsDNA. At first, we carried out the replications at 37 °C using Klenow exo-polymerase after annealing step for dsDNA templates and primer. The discrimination of 6mA from A still could be achieved, but was not as significant as single-stranded DNA (Fig. S13†). To magnify the extension difference between A and 6mA, we introduced the amplification process with cycles of denaturation, annealing and replication using DNA Taq polymerase. With the increased cycles of amplification, tenfold primers were completely extended for A containing dsDNA with the aid of Ag^+^, leaving primers for 6mA containing dsDNA almost unchanged (Fig. 5). Taking advantage of this efficient strategy, 500 pM 6mA can be discriminated from A in dsDNA (Fig. S14†), making it a potential assay for the detection of 6mA in genomic DNA.

### Metal-ion mediated replication combined with RCA

Encouraged by the high selectivity and reproducibility of our method described above, we combined this selective metal ion-mediated replication with rolling circle amplification (RCA) for signal enhancement. RCA is an isothermal and exponential amplification that can generate RCA products containing thousands of tandem DNA repeats that are complementary to the circularized padlock probe.^18,19^ This high amplification efficacy has led to its widespread use in the detection of DNA,^20^ RNA,^21^ single nucleotide polymorphisms (SNPs),^22^ metal ions^23^ and proteins.^24^ However, this experiment here is the first reported attempt to detect 6mA combined with RCA.

**Fig. 6:**
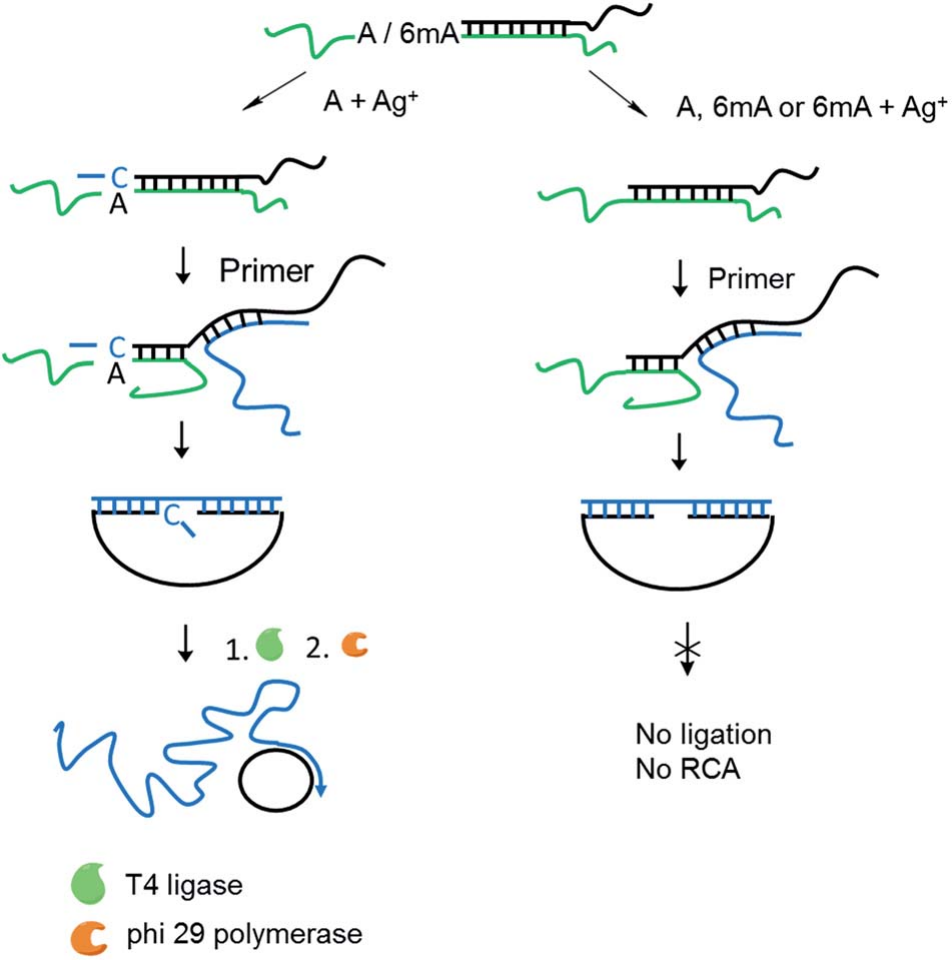
Scheme of 6mA detection through the RCA reaction based on metal ion-mediated replication. The green line represents templates containing A or 6mA. The padlock probe with a long tag (black line) acted as an extension primer here. The RCA primer (blue line) was introduced (1) to bring the two ends of the extended padlock probe together to form a circle precursor and (2) to serve as the primer in the subsequent amplification.

The 6mA detection strategy based on metal ion-mediated replication and RCA is presented in Fig. 6. The padlock probe is first designed to hybridize with the target templates. For A-containing templates (A–TA and A–GG), the subsequent Ag^+^-promoted replication would introduce one or more nucleotides into the 3′ end of the padlock probe. Then, with the addition of the RCA primer, the two ends of the extended padlock probe would be brought into proximity to form a circle precursor through toehold-mediated strand displacement, initiating the ligation by T4 DNA ligase. In contrast, for 6mA-containing templates (6mA–TA or 6mA–GG), the ligation and circularization of the padlock probe were blocked even with the addition of Ag^+^ due to incomplete complementary binding between the padlock probe and the RCA primer. The same inhibitions of ligation were also observed for A- and 6mA-containing templates without Ag^+^. Thus, only the A-containing templates with Ag^+^ can trigger the RCA reaction and yield a fluorescence signal using an intercalating dye (SYBR Green I).

**Fig. 7:**
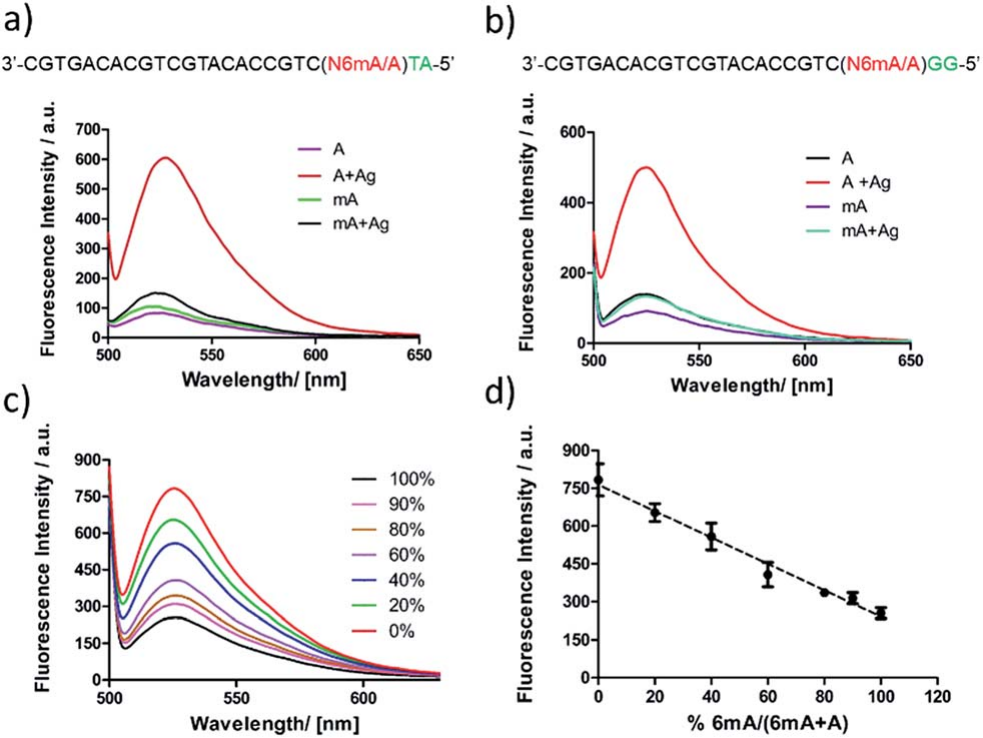
Identification and quantification of 6mA *via* metal ion-mediated replication and the RCA reaction. The discrimination of 6mA from A through fluorescence measurement using SYBR Green I for (a) templates A/6mA–TA and (b) templates A/6mA–GG. Excitation wavelength: 488 nm. (c) Fluorescence emission spectra of mixed samples with diverse proportions of methylated DNA. Here, 0% means 0% 6mA-containing templates, 100% means 100% 6mA-containing templates (d) linear relationship between fluorescence intensities and methylation levels. Error bar, mean ± SEM, *n* = 3.

First, DNA templates containing A or 6mA (A/6mA–TA) were characterized using this strategy. Without the addition of Ag^+^, both templates containing either A or 6mA have much lower fluorescence signals (Fig. 7a). Once Ag^+^ was added, however, a stable A–Ag^+^–C complex was formed for the A-containing templates (A–TA), initiating the extension and RCA reactions. As a result, we observed significant signals through fluorescence measurement for A-containing templates, successfully discriminating 6mA from A in DNA templates (Fig. 7a).

The determination of 6mA in another series of DNA templates (A/6mA–GG) was also successfully achieved using this fluorescence strategy, demonstrating negligible differences in selectivity for these two series of DNA templates (Fig. 7b). This fluorescence strategy also worked well for the quantitative evaluation of the degree of methylation in mixed samples (Fig. 7c). As shown in Fig. 7d, the fluorescence intensity was linearly proportional to the percentage of 6mA. Furthermore, the products of RCA amplification were sequenced to verify the successful incorporation of dCMP in the padlock probe (Fig. S15†).

## Conclusions

In summary, we have demonstrated the remarkable difference in stability between A–Ag^+^–C and 6mA–Ag^+^–C due to the steric hindrance caused by the methyl group on the N6 position of adenine. As only A–Ag^+^–C can be efficiently recognized by DNA polymerases, we successfully discriminated 6mA from A in both ssDNA and dsDNA at the single-base level through the analysis of extended primers. Then, combined with the RCA reaction for signal enhancement, we also identified and quantified 6mA through fluorescence measurements. The high selectivity and sensitivity of this strategy may make it useful in the future analysis of 6mA in genomic DNA. Moreover, this metal ion-mediated replication and the RCA reaction may also provide a new platform for the identification of other methylated bases, such as N4-methylcytosine (4mC), as disparate stabilities are also observed between 4mC–Ag^+^–A and C–Ag^+^–A.
