# Supplementary material for: Selective detection of N6-methyladenine in DNA via metal ion-mediated replication and rolling circle amplification
Source: Chem Sci. 2016 Aug 10;8(1):200–5. doi: 10.1039/c6sc02271e (PMC5308289; doi:10.1039/c6sc02271e)
Supplement: Supplementary file 1 [file SC-008-C6SC02271E-s001.pdf]

Electronic Supplementary Material (ESI) for Chemical Science.
This journal is © The Royal Society of Chemistry 2016

Supporting Information

Selective detection of N6-Methyladenine in DNA via Metal ion-mediated Replication and Rolling Circle Amplification **

Tingting Hong, Yushu Yuan, Tianlu Wang, Jingwei Ma, Qian Yao, Xiaoluan Hua, Yu Xia & Xiang Zhou *

College of Chemistry and Molecular Sciences, Institute of Advanced Studies, Wuhan University, Wuhan, Hubei, 430072, P. R. China

*xzhou@whu.edu.cn

Table of Contents

Experimental Methods

Table S1 Primers and templates used in this strategy.

Fig. S1 Effects of Ag^+ on the activity of KF exo- DNA polymerase.

Fig. S2 The efficiencies of dCMP incorporation for A/6mA containing templates with different incubation time.

Fig. S3 Primer extensions for A/6mA containing templates with different concentrations of Ag^+.

Fig. S4 The efficiencies of dCMP incorporation for A/6mA containing templates using increasing concentrations of KF exo- DNA polymerase.

Fig. S5 Primer extensions for A/6mA containing templates using increasing concentrations of dCTP.

Fig. S6 The efficiencies of dAMP incorporation for C, 5mC, 5hmC, 5fC and 5caC containing templates.

Fig. S7 Primer extension differences among A, 1mA, 6mA containing templates.

Fig. S8 Single nucleotide incorporation using Taq DNA polymerase.

Fig. S9 Full-length elongations for other two A/6mA containing templates using Taq DNA polymerase in the presence of 10 μM Ag^+.

Fig. S10 The effect of other metals ions compared with Ag^+ on the discrimination of 6mA from A for A/6mA-TA.

Fig. S11 The effect of other metals ions compared with Ag^+ on the discrimination of 6mA from A for A/6mA-GG.

Fig. S12 Discrimination of 6mA from A in double-stranded DNA templates using klenow exo- DNA polymerase without annealing step.

Fig. S13 Discrimination of 6mA from A at 37°C using klenow exo- polymerase after annealing the dsDNA templates and primer.

Fig. S14 Discrimination of 6mA from A in dsDNA at low concentrations using Taq DNA polymerase.

Fig. S15 Sequencing of the DNA repeats in RCA products for A-containing templates (A-TA, A-GG).

Experimental Methods

General methods. 2'-Deoxycytidine 5'-triphosphate (dCTP), 2'-Deoxythymidine 5'-triphosphate (dTTP), 2'-Deoxyguanosine 5'-triphosphate (dGTP) and dNTP mix (2.5 mM for each) were purchased from TaKaRa Biotech (China). N^4-Methyl-2'-deoxycytidine-5'-Triphosphate (N^4mdCTP), N^6-Methyl-2'-deoxyadenosine-5'-Triphosphate (N^6mdATP) were purchased from Trilink Biotechnologies Inc. Klenow Fregment (exo-), Taq DNA polymerase, T4 DNA ligase and phi29 DNA polymerase were purchased from thermo Fisher Scientific. The primers and templates used in primer extensions and RCA were purchased from TaKaRa Biotech (China) and their sequences were listed in Table S1. The extended bonds analyzed by polyacrylamide gel electrophoresis were scanned with a Pharos FX Molecular imager (Bio-Rad, USA) operated in the fluorescence mode. DNA concentrations were quantified by NanoDrop 2000c (thermo scientific, USA). Fluorescence signals were measured on PerkinElmer LS 55 (PerkinElmer, USA).

Single nucleotide incorporation reactions using KF exo- DNA polymerase for A/6mA-containing templates. The reactions were performed in a reaction buffer containing 10 mM Tris-AcOH (pH 7.9), 100 mM AcONa, 10 mM $Mg(OAc)_2$, and 5 mM NH_4Cl, 8 μM DTT. The other components used in this reaction with a final concentration were 100 nM primer, 150 nM templates, 20 μM dCTP, 10 μM Ag^+ and 0.5 Unit KF exo- DNA polymerase. The reactions were carried out at 37 °C for 30 min and were quenched by adding 20 μl formamide and 2 μl loading buffer. Then the mixtures were heated at 90 °C for 10 min immediately and cooled down to 4 °C before loaded to the 20% denaturing polyacrylamide gel. In this condition, the primer was 100% extended into 22-mer on A-containing template, while virtually no primer extension was detected on 6mA-containing template.

Single nucleotide incorporation reactions using Taq DNA polymerase. The final concentrations of the primer, templates, dCTP and Ag^+ used were the same as those used with KF exo-DNA polymerase. The reactions were carried out in 1 × Taq reaction buffer containing 10 mM Tris-HCl (pH 8.8), 50 mM KCl and 2.5 mM $MgCl_2$ in the presence of 2 Unit Taq DNA polymerase at 68 °C for 1 h. After incubation, the reaction was quenched and analyzed by 20% denaturing PAGE.

Discrimination of 6mA from A in double-stranded DNA templates using Taq DNA polymerase. The reactions with 1μM FAM labeled primer, 100 nM A or 6mA containing double-stranded DNA templates, 40 μM dCTP and 20 μM Ag^+ were performed in 1 × Taq reaction buffer containing 10 mM Tris-HCl (pH 8.8), 50 mM KCl and 2.5 mM $MgCl_2$ in the presence of 1 Unit Taq DNA polymerase. The replications were carried out under the following conditions: 95 °C for 3 min, and then 10-20 cycles at 95 °C for 15s and 68 °C for 10min. After incubation, the reactions were immediately quenched with formamide and heated at 90 °C for 10 min, followed by analysis through 20% denaturing PAGE.

Metal ion-mediated replication combined with rolling circle amplification. For the primer extensions, 6 nM padlock probe was extended in the prescence of 5 nM templates containing A/6mA, 1 μM Ag^+, 10 μM dCTP and 0.25 Unit KF exo- in the reaction buffer containing 10 mM Tris-AcOH (pH 7.9), 100 mM AcONa, 10 mM $Mg(OAc)_2$, and 5 mM NH_4Cl, 8 μM DTT. After incubation at 37 °C for 5 min, the reaction was terminated by heating at 70 °C for 10 min. Then, 7.5 nM RCA primer was added to hybridize with the padlock probe through toehold-mediated strand displacement. The circle precursor padlock was ligated by using 2.5 Unit T4 DNA ligase at 22 °C for 2h. The RCA reaction was performed at 30 °C for 6 h by adding 1 × phi 29 reaction buffer, 200 μM dNTP, 3 Unit phi 29 DNA polymerase. For the fluorescence measurement, the RCA products were mixted with 5 μl 20 × SYBR green I and diluted to a final volume of 200 μl.

Table S1 Primers and templates used in this strategy.

Name	Sequence (5' to 3')
Primer-21mer	FAM-GCA CTG TGC AGC ATG TGG CAG
Primer-22mer	FAM-GCA CTG TGC AGC ATG TGG CAG C
Primer-24mer	FAM-GCA CTG TGC AGC ATG TGG CAG CAG G
A/6mA-TA	AT*6mA/A* CTG CCA CAT GCT GCA CAG TGC
A/6mA-GG	GG*6mA/A* CTG CCA CAT GCT GCA CAG TGC
A/6mA-GC	CG*6mA/A* CTG CCA CAT GCT GCA CAG TGC
Padlock probe	PO_4- [illegible]
RCA primer-G	TTCGGGACTGACGGCGCTGCCACATGCTGCACCCTACTCCC
RCA primer-GGG	TTCGGGACTGACGGCGGGCTGCCACATGCTGCACCCTACTCCC

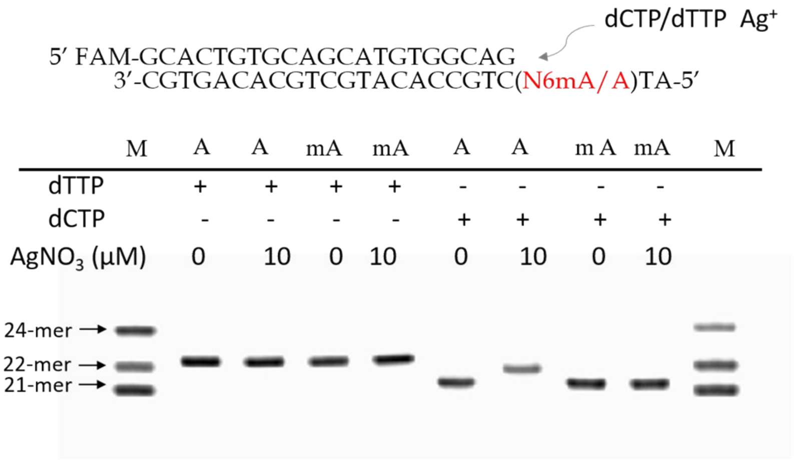

Fig. S1 Effects of Ag^+ on the activity of KF exo- DNA polymerase. In the presence of dTTP, primers were all efficiently extended into 22-mer with or without Ag^+ for both A and 6mA-containing templates, indicating the high activity of KF exo- DNA polymerase even in the presence of Ag^+. While with the addition of dCTP, only the sample containing Ag^+ and template-A can produce extended primers.

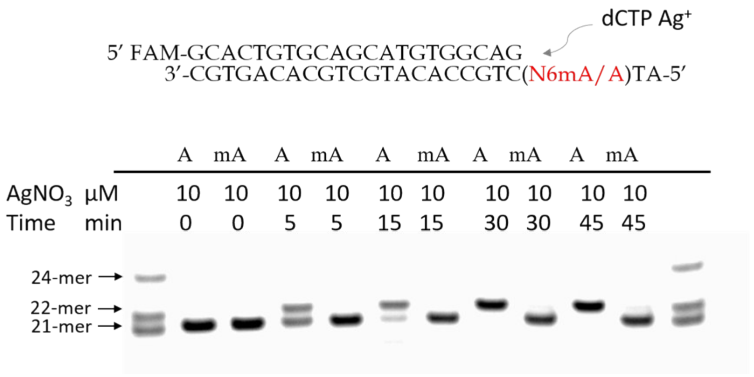

Fig. S2 The efficiencies of dCMP incorporation for A/6mA containing templates with different incubation time. The 21-mer primers were 100% extended into 22-mer after 30 min incubation at 37 °C for A-containing templates. The primer extension was still terminated after 45 min incubation. M indicates the marker for the 21-mer, 22-mer and 24-mer primers.

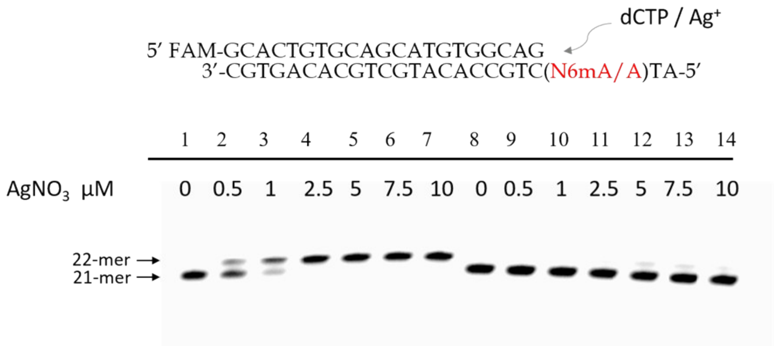

Fig. S3 Primer extensions for A/6mA containing templates with different concentrations of Ag^+. Lane 1-7, 21-mer primers were gradually extended into 22-mer with the increased concentrations of Ag^+ (from 0-10 μM) for A containing templated. Lane 8-14, virtually no primer extensions were detected for 6mA containing templates.

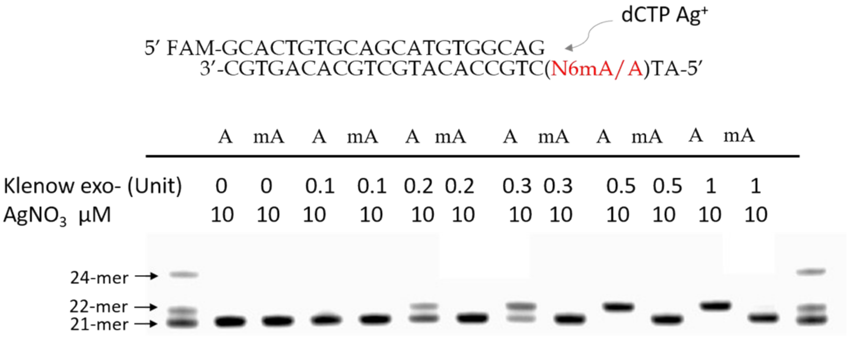

Fig. S4 The efficiencies of dCMP incorporation for A/6mA containing templates using increasing concentrations of KF exo- DNA polymerase. The reactions were performed under the condition described above. For A-containing templates, the 21-mer primers were gradually extended into 22-mer by adding increased concentrations of KF exo- DNA polymerase (from 0 Unit to 1 Unit). For 6mA-containing templates, virtually no extended primers were detected, showing striking difference with A-containing templates. M indicates the marker for the 21-mer, 22-mer and 24-mer primers.

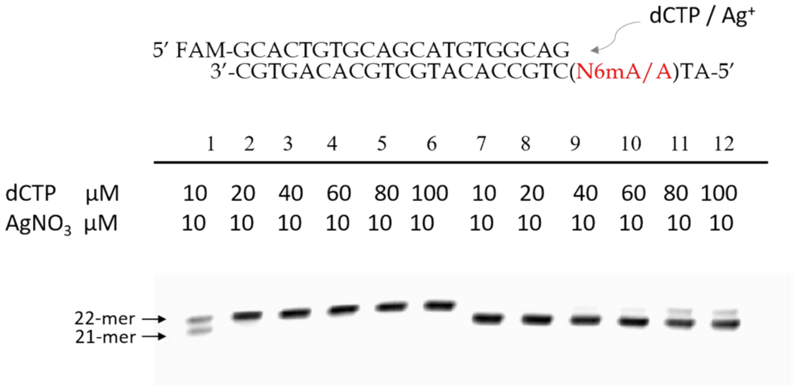

Fig. S5 Primer extensions for A/6mA containing templates using increasing concentrations of dCTP. The reactions were performed under the condition described above. Lane 1-6, A containing DNA were used as templates and the 21-mer primers were totally extended into 22-mer for 20 μM dCTP or more. Lane 7-12, 6mA containing DNA were used as templates, virtually no primer extensions were observed for 10 μM, 20 μM, 40 μM and 60 μM dCTP. And even with the further increased concentration of dCTP (80 μM and 100 μM), much fewer extended primers were detected for 6mA containing templates compared with A containing templates.

a)

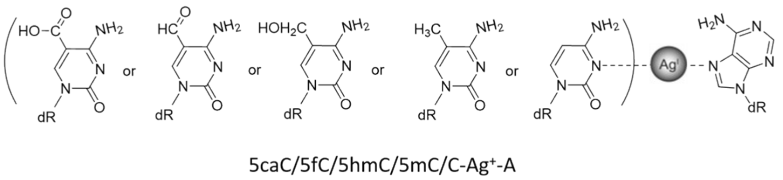

b)

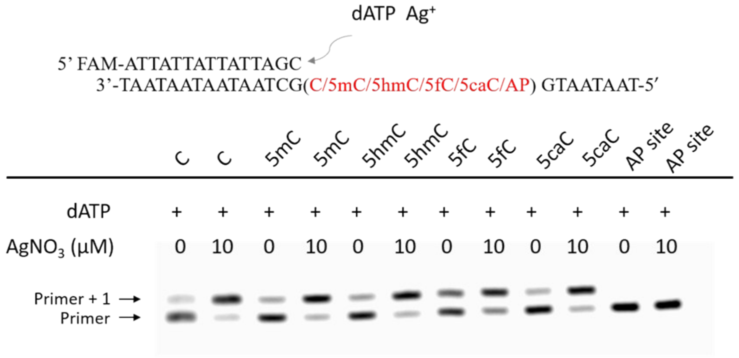

c)

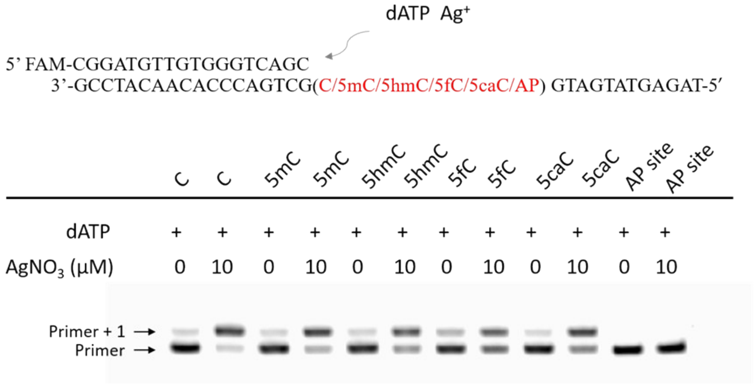

Fig. S6 The efficiencies of dAMP incorporation for C, 5mC, 5hmC, 5fC and 5caC containing templates. a) Structures of Ag^+ stabilized base pairs (C/5mC/5hmC/5fC/5caC-Ag^+-A). Sequences of b) series 1 and c) series 2 and corresponding primer extensions with the addition of Ag^+.

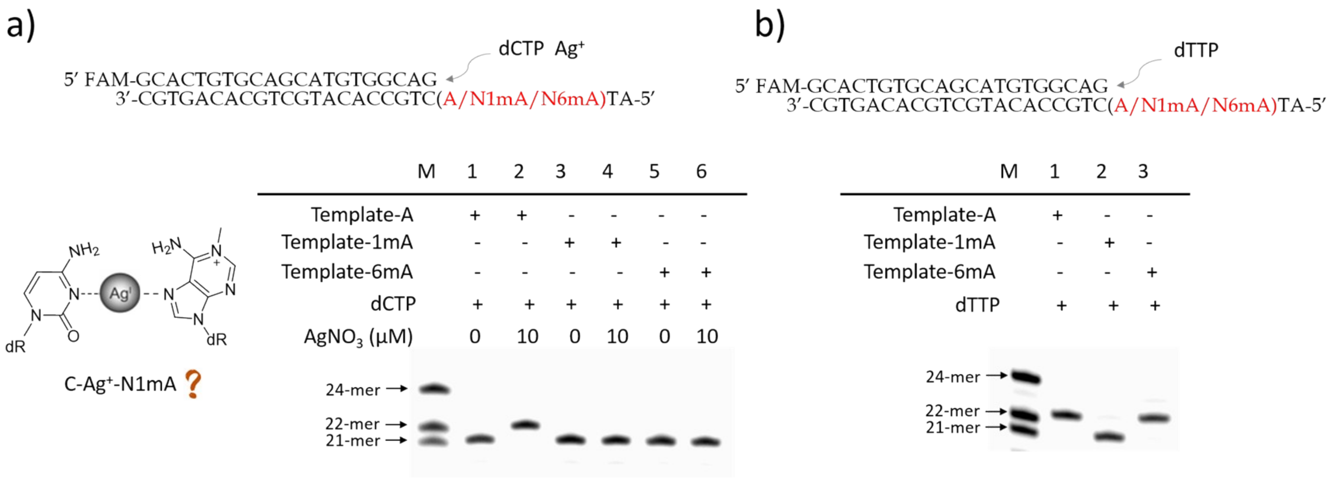

Fig. S7 Primer extension differences among A, 1mA, 6mA containing templates. a) Incorporation efficiencies of dCMP opposite A/N1mA/N6mA residues in DNA templates in the absence or presence of Ag^+. The reactions were performed under the same conditions described above. b) Primer extensions for A/N1mA/N6mA containing templates in the presence of dTTP using klenow exo-. The reaction time was 5 min at 37°C. The extension was only terminated for 1mA containing template.

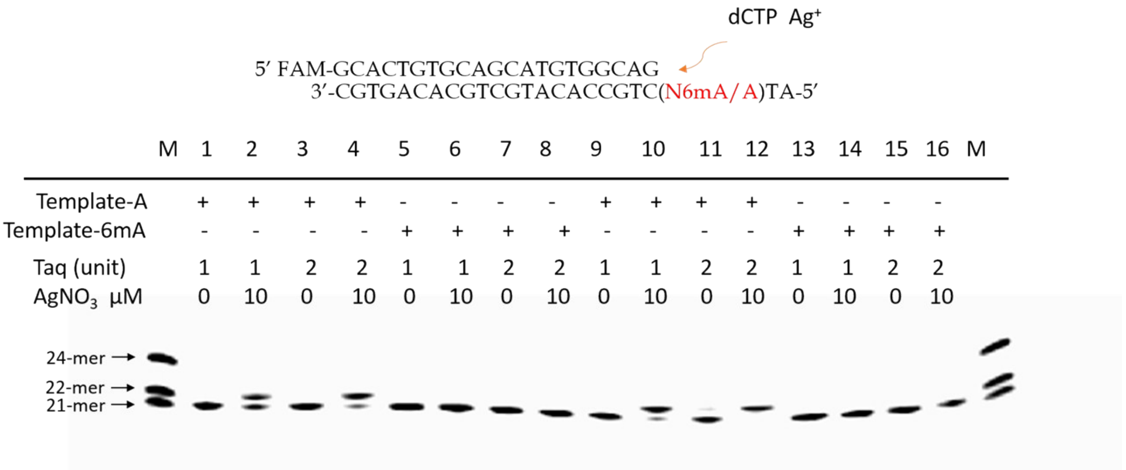

Fig. S8 Single nucleotide incorporation using Taq DNA polymerase. Lane 1-4 and Lane 9-12, A-containing DNA were used as templates; Lane 5-8 and Lane 13-16, m6A-containing DNA were used as templates. The reaction time for Lane 1-8 and Lane 9-16 was 30 min and 1 h, respectively. The data showed that dCMP can be also efficiently incorporated into primers with the aid of Ag^+ for A-containing templates, while for 6mA-containing templates, even 2 Unit Taq DNA polymerase with 1 h incubation cannot catalyze the primer extension. M indicates the marker for the 21-mer, 22-mer and 24-mer primers.

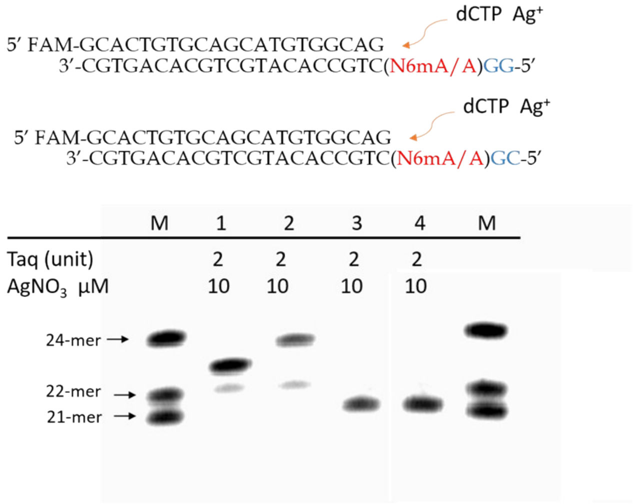

Fig. S9 Full-length elongations for other two A/6mA containing templates using Taq DNA polymerase in the presence of 10 μM Ag^{+}. The template used in Lane 1-4 was A-GC, A-GG, 6mA-GC, and 6mA-GG, respectively. The introduction of Ag^{+} stabilized A-C mispair did not affect the full-length elongation for A-containing templates.

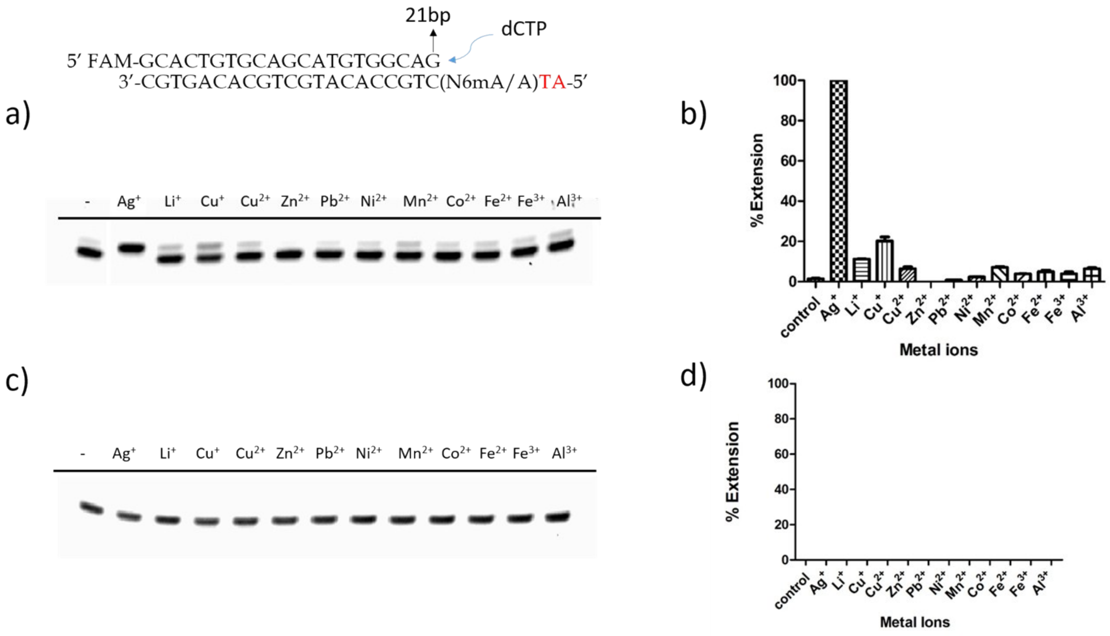

Fig. S10 The effect of other metals ions compared with Ag^{+} on the discrimination of 6mA from A for A/6mA-TA. a) and b) are the PAGE analysis and relative extension percentages of single nucleotide incorporation using various metal ions for A-TA templates. c) and d) are the PAGE analysis and relative extension percentages of single nucleotide incorporation using various metal ions for 6mA-TA templates. The reactions were performed under the same conditions described above in the presence of 10 μM metal ions.

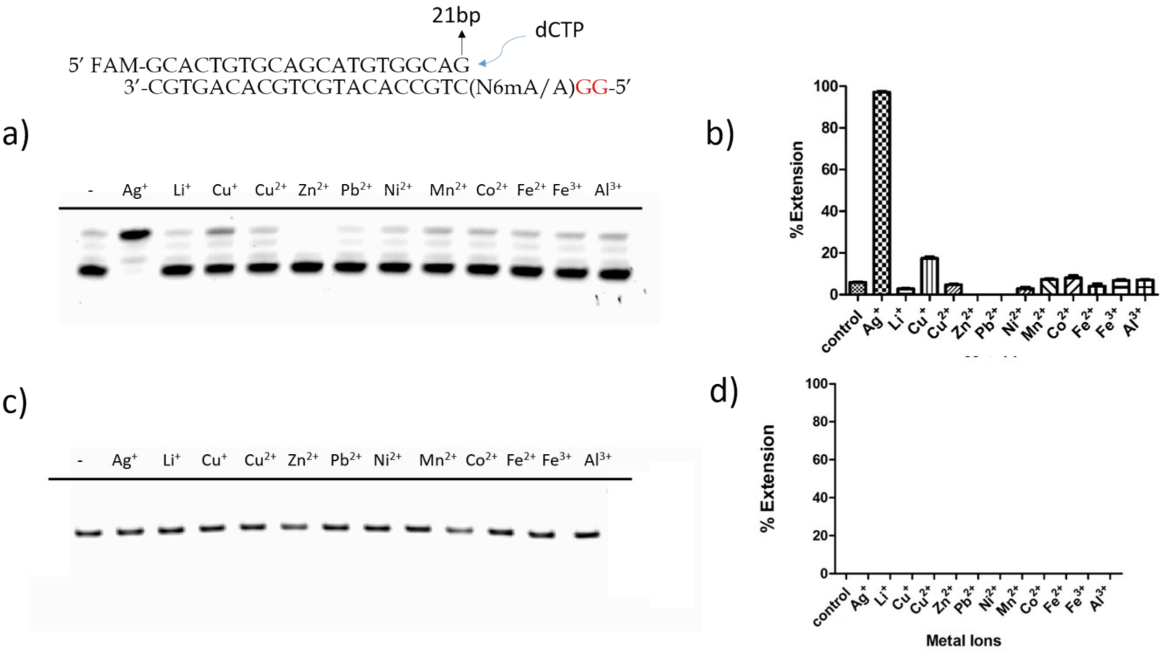

Fig. S11 The effect of other metals ions compared with Ag^+ on the discrimination of 6mA from A for A/6mA-GG. a) and b) are the PAGE analysis and relative extension percentages of the full-length extensions using various metal ions for A-GG templates. c) and d) are the PAGE analysis and relative extension percentages of the full-length extensions using various metal ions for 6mA-GG templates. The reactions were performed under the same conditions described above in the presence of 10 μM metal ions.

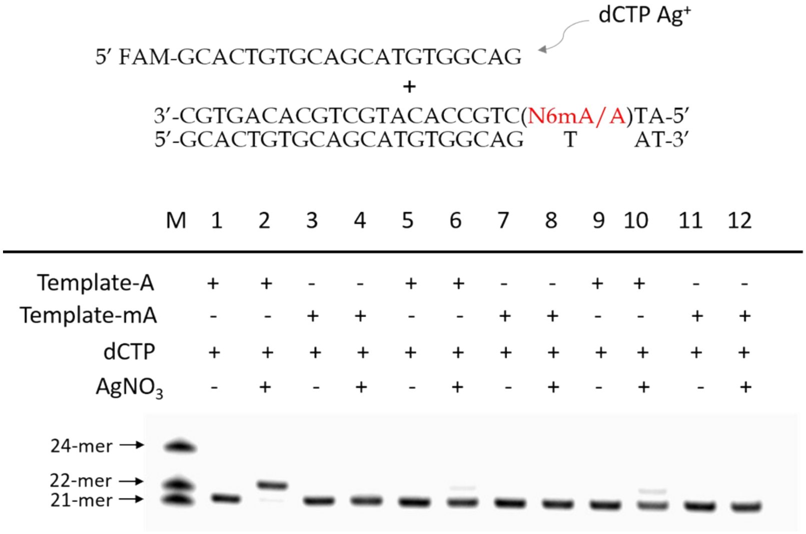

Fig. S12 Discrimination of 6mA from A in double-stranded DNA templates using klenow exo- DNA polymerase without annealing step. Lane 1-4, the Ag^+ mediated replication worked well for the discrimination of 6mA from A in single-stranded DNA templates. While little difference was observed between 6mA and A in double-stranded DNA even with the addition of Ag^+. The incubation time with klenow exo- DNA polymerase at 37°C was 30 min and 2 h for Lane 5-8 and Lane 9-12, respectively.

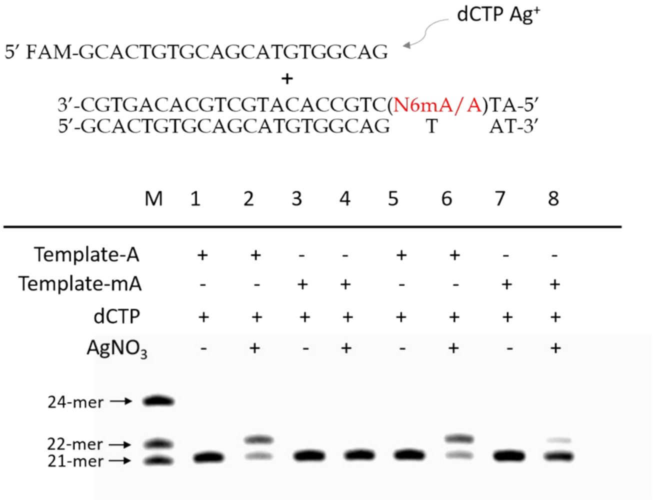

Fig. S13 Discrimination of 6mA from A at 37°C using klenow exo- polymerase after annealing the dsDNA templates and primer. The reaction time was 30 min and 1 h for Lane 1-4 and Lane 5-8, respectively. The primer and dsDNA template were heated to 95°C for 5 min and then were slowly cooled down to room temperature before the replication at 37°C. The primer extension was much more efficient than that without annealing step (Fig. S12), but was still not as high as ssDNA even after 1 h incubation.

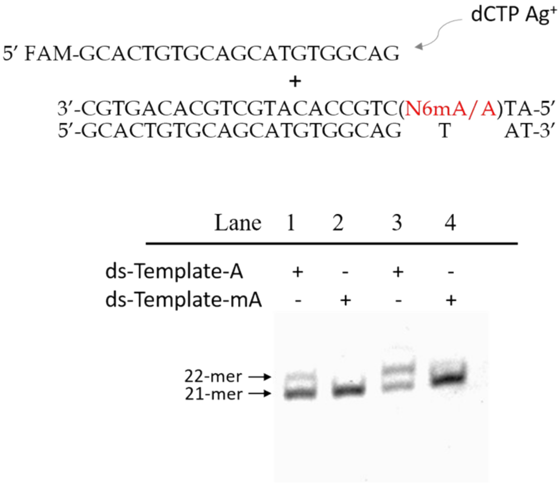

Fig. S14 Discrimination of 6mA from A in dsDNA at low concentrations using Taq DNA polymerase. The concentration of Lane 1-2 and Lane 3-4 was 500 pM and 1 nM, respectively. The primer was 10 nM and the amplification reactions were performed through 15 cycles of denaturation at 95°C for 15s, annealing and replication at 68 °C for 10 min.

a)

b)

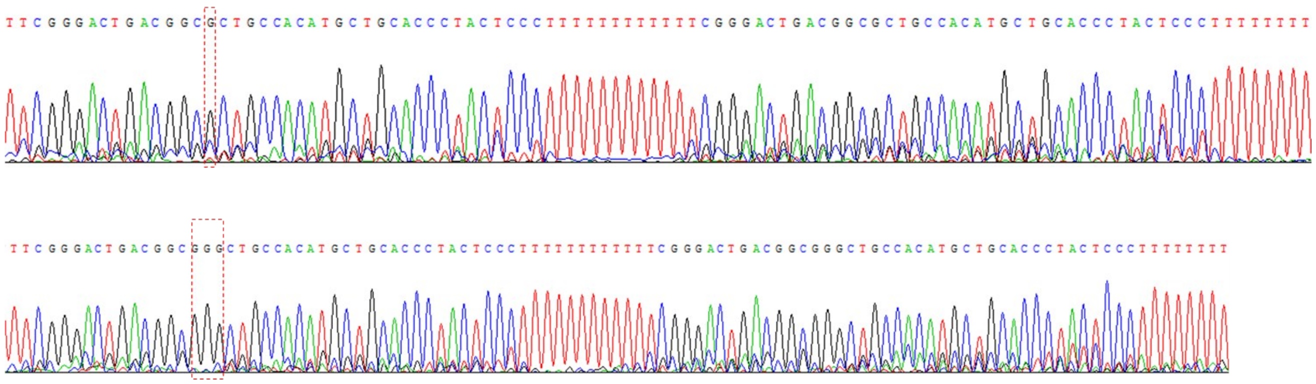

Fig. S15 Sequencing of the DNA repeats in RCA products for A-containing templates a) A-TA, b) A-GG. The highlighted Guanine in red boxes were complementary to the cytosine introduced by Ag^+ mediated replication in the circularized padlock probes.
